# Chit-Chat

**Published:** 1881-01

**Authors:** 


### Editorial Chit-Chat

NEW TYPE.—The next issue of the BISTOURY, commencing a new volume, will appear dressed in new type and heavier paper.

BOUND VOLUMES.—The BISTOURY, neatly bound, and indexed, foı past three years, in one volume, sent post paid on receipt of $2.50.

CROWDED OUT.—A portion of Correspondence—extremely spicy it is, too—and our Household Hints and Recipes must lie over until next time, being crowded out of their usual position to make room for the Index.

AMERICAN SOCIETY OF MICROSCOPISTS.—It is not improbable that the next meeting of this scientific society will occur in Elmira. An invitation has been extended by the society in this city, and, from the correspondence now transpiring, it seems quite likely that Elmira will be honored by a visitation of all the distinguished microscopists of the country, next August.

"SATISFACTORY SETTLEMENT."—It is a sad commentary upon the integrity of the average life insurance company that now and then induces them to publish to the world the startling circumstance that they have made a "satisfactory settlement" of a claim against them. Why should they not settle satisfactorily? What do men pay their money for, if not for satisfactory settlement?

The truth of the matter is, and one that seems to be recognized by the companies mentioned, a "satisfactory settlement" is so rare an occurrence upon *their* part, that it is worth hurrahing about when they see no means of wiggeling out of it. It has come to pass that the insured party is too often forced into an expensive lawsuit, or compelled to take an enormous reduction upon his claim to prevent one; and a quiet, honest, "satisfactory settlement" is a rare, therefore noteworthy proceeding.

THE KLAN.—A society of gentlemen who take turns in giving competitive luncheons once in two weeks, have commenced their winter campaign. Two meetings have so far taken place, and the survivors are patiently but anxiously waiting for a wild turkey to drop that has been undergoing the ripening process since thanksgiving day. The delicacy of taste exhibited by this society of epicures is something to be envious of, and the criticisms pronounced upon the feasts, an exhibition of wonderful familiarity with the refinements of cooking.

The meetings would be held more frequently were it not for the circumstance that it requires about that time for the fellows to recover wholly from the effects of their last luncheon, before they are able to take in another.

SARA BERNHARDT.—We are glad to note that this distinguished woman has been received by the American people in a manner highly creditable both to the actress and her patrons. While the actress has deilghted her audiences and received ample applause and remuneration in return, the frailties of the woman as personified in Miss Bernhardt have not been countenanced nor applauded, as was the case among the nobility of England. It is one thing to recognize the artiste, quite another to approve of sins committed against the usages of society.

While society has administered a rebuke to Sara's frailties, the actress herself has not been slow to administer well deserved censure to her assailants, in return. "What! several children and no husband!" exclaims the fashionable woman. "What! several husbands and no children!" retorts Sara. To those having a regard for the decencies of society, the two situations may be regarded as a stand-off.

THE IRISH QUESTION—is something of a poser to us. There seems to be no doubt that Irish tenants are often illy and perhaps unmercifully treated by their English landlords. It would seem also, that the landlords are not without cause for complaint against the tenants. To interfere with a man's working his own farm and selling the products thereof whenever and whereever he chooses, is a piece of highanded communism that would not be tolerated in this country. Doubtless, it was wrong to permit the nobility of England to own nearly all of the Irish soil. It would have been much better for both parties if the land could be divided among the peasantry. But just how that can be done now, without working great loss to the owners of the land, does not seem so clear. The condition of affairs in Ireland should practically demonstrate the great danger to our own country in giving immense tracts of western lands to railroad or other powerful corporations. This practice, by Congress, should be at once abolished, and our public lands sold only to actual settlers or those contemplating settling upon them. By pursuing this course, we may one day see the wisdom of it, and rejoice that we have escaped a difficulty that has placed England in a predicament that threatens the stability of her government.

---

CREMATION AT WARREN, PA.—Recently, the cremation of the body of a very estimable lady of Warren, Pa., occurred, [according to her expressed desire to her husband, in her lifetime,] at the Crematory at Washington, Pa. A brother of the lady, who witnessed the incineration of the body, describes the process as being a cleanly and really beautiful method of disposing of the dead. Not the slightest thing occurred to shock the feelings of the most sensitive nature, he declares, and has avowed his intention of leaving directions to have his own body disposed of in like manner.

We are quite sure that, if a cremation furnace could be found in every city of this size, the sensible portion of community would speedily avail themselves of this cleanly and wholesome method, rather than resort to the heathenish and polluting one of inhumation.

---

THE BEECHER SUPPER.—Soon after the results of the election had been made known, a few of the friends of the Rev. Thomas K. Beecher assembled and determined to give him a congratulatory supper. It seemed manifest that two reasons for congratulation existed: One, that Mr. Beecher should have made such an unlooked for run—polling more votes, in his own county, than was ever given any candidate before, and coming within a few hundred votes of being elected to Congress—and the other, that he was permitted to remain at home among the people who love him so dearly and respect him so highly. Mr. Beecher was nominated by a party hopelessly in the minority; yet, without any one to "work" for him, and flatly refusing to do anything for himself, he came very near defeating the candidate who was before elected by over 5,000 majority. This was a matter for congratulation—all around—it was thought; hence the supper. The table was spread, on a Monday evening, at Haight's hotel, where about thirty of Mr. Beecher's friends—of all political complexions—were assembled to do him honor. It seemed as though all the witty fellows in the city, capable of making an after-supper speech, were there. It is safe to say that a more sparkling supper—without the aid of wine—was never sat down to in this city. The toast-master could not have been better selected, for he brought out the characteristics of the party, in response to his admirably arranged themes, in a manner truly refreshing. The affair was well conceived, admirably carried out and a pronounced success in every particular.

---

END OF VOLUME XVI.—This number closes the volume as well as the year. By reference to the very full and complete index which accompanies this number, it will be seen that we have afforded a vast amount of information and instruction at exceedingly small cost. Very many of our subscribers are far in arrears with their payments to the BISTOURY. The sum is so small that to make special application for it by letter, would not pay for the trouble incurred. We call upon *all* who receive this number of the BISTOURY, to remit the subscription price promptly. If the fifty cents is not convenient, forward a dollar note, when you will be duly credited for two years.

To those who habitually neglect to remit, we can only say a day of reckoning will come, when the sum reaches sufficient proportions to warrant a compulsory collection.

---

ROCHESTER OPTICIANS.—The President and the Secretary of the Elmira Microscopical Society recently made a visitation to several noted opticians and microscopists, and spent a day with those at Rochester. They report a very interesting time with Gundlach—the famous microscopic objective maker, who has just produced a wonderful one-eighth, of wide angular aperture and immense working distance. Two of these somewhat famous objectives are owned in Elmira, and are pronounced to be "the best in the world."

They also visited the extensive optical works of Bausch & Lomb, and speak highly of the courtesy shown them by Mr. Edward Bausch, who at sacrifice of much valuable time, accompanied them over their extensive establishment, explaining the curious and complicated machinery used in the manufacture of spectacles and eye-glasses. More than one hundred and fifty men and women were found employed in the construction of these articles. The labor-saving machines employed in the construction of frames and lenses were truly wonderful. It would seem that this firm are turning out, monthly, enough spectacles to supply every man, woman and child on the habitable globe.

They manufacture also fine microscope stands and objectives, and exhibited beautiful specimens of their handiwork in this department. The company, having devised tools and machinery for the construction of microscopes, are able to supply very satisfactory stands at an exceedingly small cost.

The visiting party report that they have seen numerous establishments for the manufacture of optical goods, but none that can compare either in the magnitude of the works nor in quality of goods produced, with the Bausch and Lomb Optical Company.

---

A SKATING RINK NEEDED.—No more healthful exercise could be invented for the young people than that of skating. Just now, they are having a royal time upon the ice. But the continually appearing snow storms will soon bury their favorite skating resorts so deep as to make out-door skating impossible.

It would seem that our city is large enough to support a skating rink,—a place where our young folks can congregate and enjoy this exhilerating pastime despite the snow and rain. We are sure that if some interprising parties would construct such a place at some convenient point in the city, it would be abundantly patronized both winter and summer. The roller skate has reached that perfection as to be quite as desirable as those of the steel blade, and making it possible for skaters to exercise no matter what the condition of the weather may be. Let us have a skating rink.

---

CHRISTMAS PRESENTS.—Now are the various members of the household anxiously consulting with one another—on the sly, as to what Santa Claus had better bring to father, mother, the children and the baby. The festal time of year has once more come to us with all its joyous features. It is well that father, mother, and children should remember each the other in some token of affection usually presented upon Christmas day. Especially should the little ones be remembered, and as abundantly too as their little hearts could wish for. Their hopes for and confidence in old Santa Claus should never be shaken, nor his mystic hiding place ever divulged. No sweeter dream comes to the little darlings during the weary days and nights now passing until the night for hanging up the tiny stockings at last arrives. Let them not hope in vain, but be sure to aid the mythical old gentleman in cramming the stockings full.

With the older members of the family, only such gifts should be exchanged as are really useful and needed. Give not for effect or ostentatiously, that neighbors may be brought to think you generous and envy you the ability to give. Give no costly gifts to friends—it does'nt pay. Usually, it is expected that the favor will be returned, or you feel that it *should* be, which is much the same, and really forces you to pay for your own present. The habit of sending gifts to acquaintances, therefore, is liable to great abuse and rarely gives the pleasure that was anticipated. Would you remember a friend on Christmas morning ? Send him or her a boquet, a Christmas card and a hearty greeting. This will be a sufficient reminder of your continued friendship and good will and may spare the friend the pang of being indebted to you for a gift that he can illy afford to repay.

---

THE BISTOURY. Edited by Thad S. Up de Graff, M. D., Elmira, N. Y. This little quarterly illustrates the possibility of the publication of a journal at once valuable to the laity and not entirely insipid to the profession. It is a somewhat difficult matter to conduct such a journal, and Dr. Graff, or de Graff, or Up de Graff (we don't know exactly where his name begins) deserves credit for the success of his effort. The BISTOURY used to keep us posted in regard to the progress of a monument which a number of his lineal descendants in Elmira were said to be erecting to the memory of a gentleman by the name of Adam (just Adam, no surname). Latterly we have heard nothing of the matter. Has work been discontinued on the stately pile ? Waiting for an appropriation, or what ?—*Therapeautic Gazette.*

Neither, dear *Gazette.* Work has not been discontinued—not by any means. Contrawise, the association now holds weekly meetings, and are patiently awaiting the completion of a design for the proposed bronze statue of a primitive man, to the unveiling of which the *Therapeutic Gazette* editors will receive cordial invitations. As to waiting for an appropriation—not at all. To assure the *Gazette* of the sound financial condition of the association, we impart a little secret, to wit: *one* person enthusiastically engaged in the enterprise, recently gave $2,500 toward the fund. Possess your soul in patience—the monument will be erected in due time, and will do credit to the filial affection, enterprise, and artistic taste of the citizens of Elmira.

